# Supplementary material for: New Cyclam-Based Fe(III) Complexes Coatings Targeting Cobetia marina Biofilms
Source: Molecules. 2025 Feb 16;30(4):917. doi: 10.3390/molecules30040917 (PMC11858526; doi:10.3390/molecules30040917)
Supplement: Supplementary file 1 [file molecules-30-00917-s001.zip › Supplementary Material_7fev proofread.docx.final.pdf]

Supplementary Material

1. Synthetic procedures for the preparation of cyclam-based iron(III) complexes

Complexes [{H_2($^{4-CF_3}$PhCH_2)$_2$Cyclam}FeCl_2]Cl, **FeCy-1**, and [{($HOCH_2CH_2CH_2$)$_2$($^{4-CF_3}$PhCH_2)$_2$Cyclam}FeCl_2]Cl, **FeCy-2**, were prepared according to previously published procedures as shown in Scheme S1 [16,21,40].

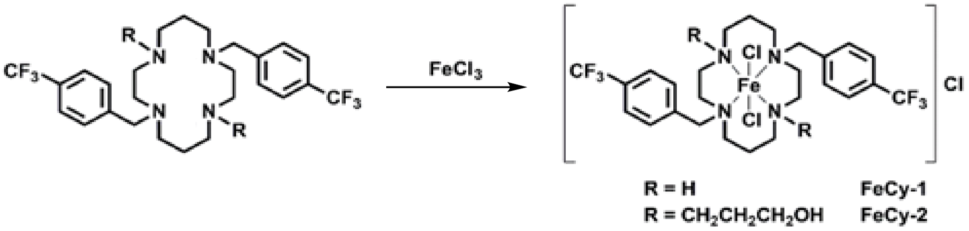

Scheme S1. Synthetic procedure for the preparation of [{H_2($^{4-CF_3}$PhCH_2)$_2$Cyclam}FeCl_2]Cl, **FeCy-1**, and [{($HOCH_2CH_2CH_2$)$_2$($^{4-CF_3}$PhCH_2)$_2$Cyclam}FeCl_2]Cl, **FeCy-2**.

FeCy-1 [16]: H_2($^{4-CF_3}$PhCH_2)$_2$Cyclam [40] (0.50 g, 0.97 mmol) was dissolved in 40 mL of methanol and a 10 mL solution of $FeCl_3$ (0.16 g, 0.97 mmol) in methanol was added. The reaction mixture was stirred at room temperature for 4 h. The solution was filtered and evaporated to dryness. The crude was washed with small portions of diethyl ether and dried under reduced pressure. The product was obtained as a yellow powder in 89% yield (0.59 g, 0.87 mmol).

FeCy-2 [21]: ($HOCH_2CH_2CH_2$)$_2$($^{4-CF_3}$PhCH_2)$_2$Cyclam [21] (300 mg, 0.47 mmol) was dissolved in a mixture of methanol/chloroform (5:1) and $FeCl_3$ (77 mg, 0.47 mmol, 1 equiv.) was added. The precipitate was separated by filtration, washed with small portions of n-hexane, and dried under reduced pressure. The product was obtained as an orange powder in 89% yield (421 mg, 0.53 mmol, 89%).

2. Flow cytometric dot-plots of *C. marina* biofilm cells grown on coatings

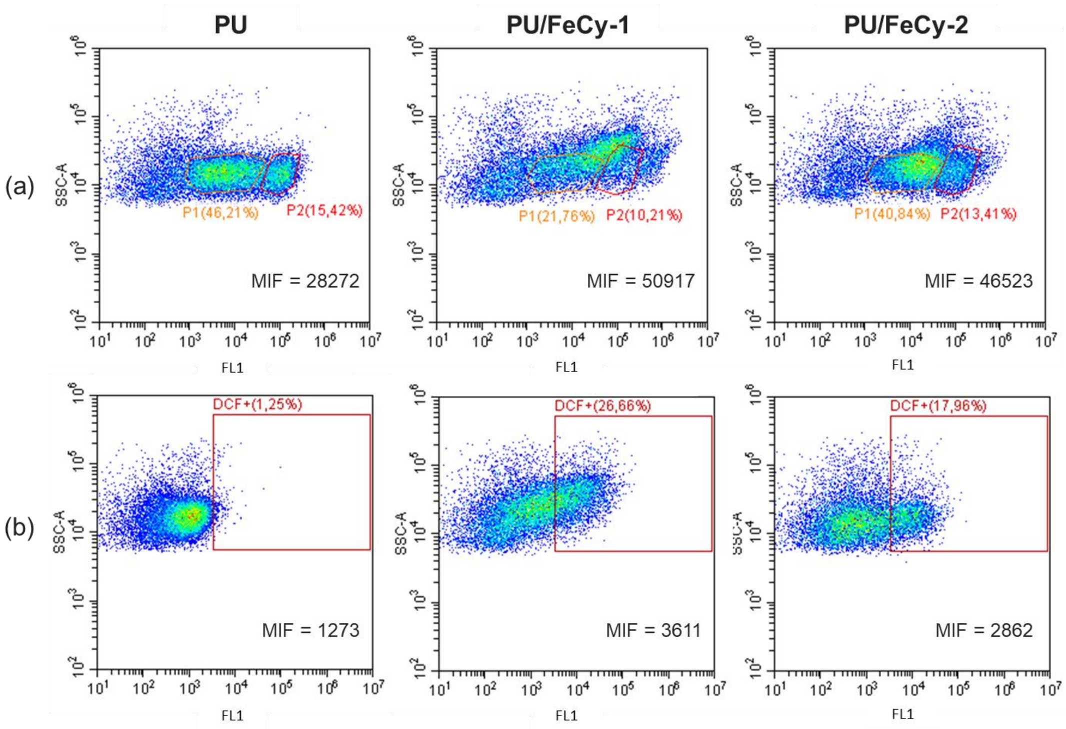

Figure S1. Representative flow cytometric dot-plots of *C. marina* biofilm cells grown on **PU** (control surface), **PU/FeCy-1** and **PU/FeCy-2** surfaces for 24 h and stained with (**a**) 5-CFDA (a metabolic activity marker) and (**b**) DCFH-DA (a reactive oxygen species (ROS) indicator). (**a**) Biofilm cells grown on the **PU** surface exhibited two populations, denoted as P1 and P2. These cell populations shifted in fluorescence (FL1 (525/40 nm)) when exposed to either **PU/FeCy-1** or **PU/FeCy-2**, resulting in an increase in mean intensity of fluorescence (MIF) and indicating changes in their metabolic activity. (**b**) Biofilm cells grown on **PU/FeCy-1** or **PU/FeCy-2** exhibited a higher percentage of DCF-positive cells (DCF+) compared to those grown on the **PU** control surface. Consequently, there was an increase in MIF of cells exposed to the PU-modified surfaces, indicating ROS production.
